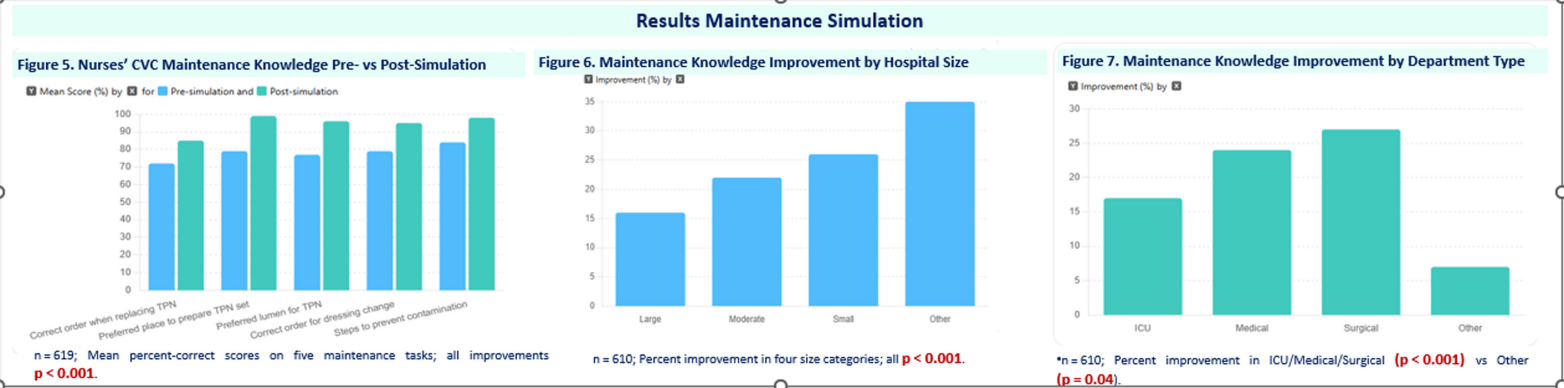# 269 System Impact and Yield of Routine Pulmonary TB Rule-Out in Extrapulmonary TB Evaluation: Isolation, Costs, and PPE Utilization

**DOI:** 10.1017/ash.2026.10632

**Published:** 2026-06-23

**Authors:** Dafna Chen, Yehuda Carmeli, Debby Ben David

**Affiliations:** 1 National Center for Infection Prevention and Antimicrobial Resistance, Israeli Ministry of Health; 2 Tel Aviv Sourasky Medical Center; 3 Wolfson Medical Center

## Abstract

**Background:** central line–associated bloodstream infections (clabsis) remain a leading cause of preventable morbidity and mortality in hospitalized patients. although evidence-based guidelines for central venous catheter (cvc) insertion and maintenance are well established, variability in knowledge, procedural practice, and access to essential equipment persists across hospitals and clinical settings. **Methods:** between january 2019 and march 2020, a mobile classroom simulation-based training (sbt) program was implemented in 26 of 30 public acute-care hospitals in israel, representing a nationwide large-scale initiative. the program combined standardized curricula, hands-on simulation, and structured feedback for cvc insertion (physicians and nurses) and maintenance (nurses). for physicians undergoing cvc insertion training, the program also included a dedicated hands-on workshop focused on ultrasound-guided central venous catheter insertion. training sessions were video-recorded using a closed internal system, allowing participants to review their performance. each trainee completed two standardized simulation scenarios, enabling real-time feedback, iterative improvement, and experiential learning. of approximately 1,700 healthcare workers who participated in the program, 1,551 (91.2%) completed both pre- and post-training questionnaires. questionnaires assessed procedural knowledge, satisfaction, and availability of cvc-related equipment. knowledge outcomes were compared using paired t-tests, and associations between hospital characteristics, equipment availability, and knowledge improvement were evaluated using chi-square analyses. **Result:** knowledge scores improved significantly across all participant groups (p<0.001). mean knowledge gains were 18.6% for physician cvc insertion, 26.5% for nurse insertion, and 16.4% for nurse maintenance. the largest improvements were observed in sterile technique, minimal barrier precautions, and maintenance-related clinical decision-making. higher baseline knowledge was associated with icu settings, larger hospitals, and availability of full personal protective equipment (ppe) kits and disinfectant wipes (p<0.05). knowledge improvement was independently associated with refresher training, structured feedback during simulation, and access to full ppe kits, whereas hospital size and professional seniority were not consistently associated with learning gains. substantial variability in availability of essential cvc equipment was observed across departments. **Conclusion:** a nationally coordinated simulation-based training program significantly improved cvc insertion and maintenance knowledge among physicians and nurses across diverse hospital settings. structured feedback and reliable access to essential equipment emerged as key drivers of learning gains. these findings support simulation-based training as a scalable, policy-relevant strategy for strengthening clabsi prevention and standardizing practice at the national level, particularly in smaller hospitals and non-icu settings. background: central line–associated bloodstream infections (clabsis) remain a leading cause of preventable morbidity and mortality in hospitalized patients. although evidence-based guidelines for central venous catheter (cvc) insertion

**Figure:**
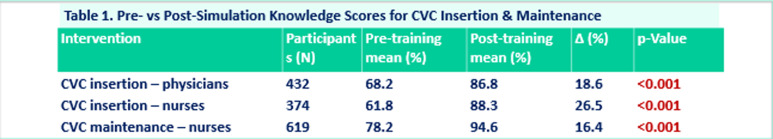

**Figure:**
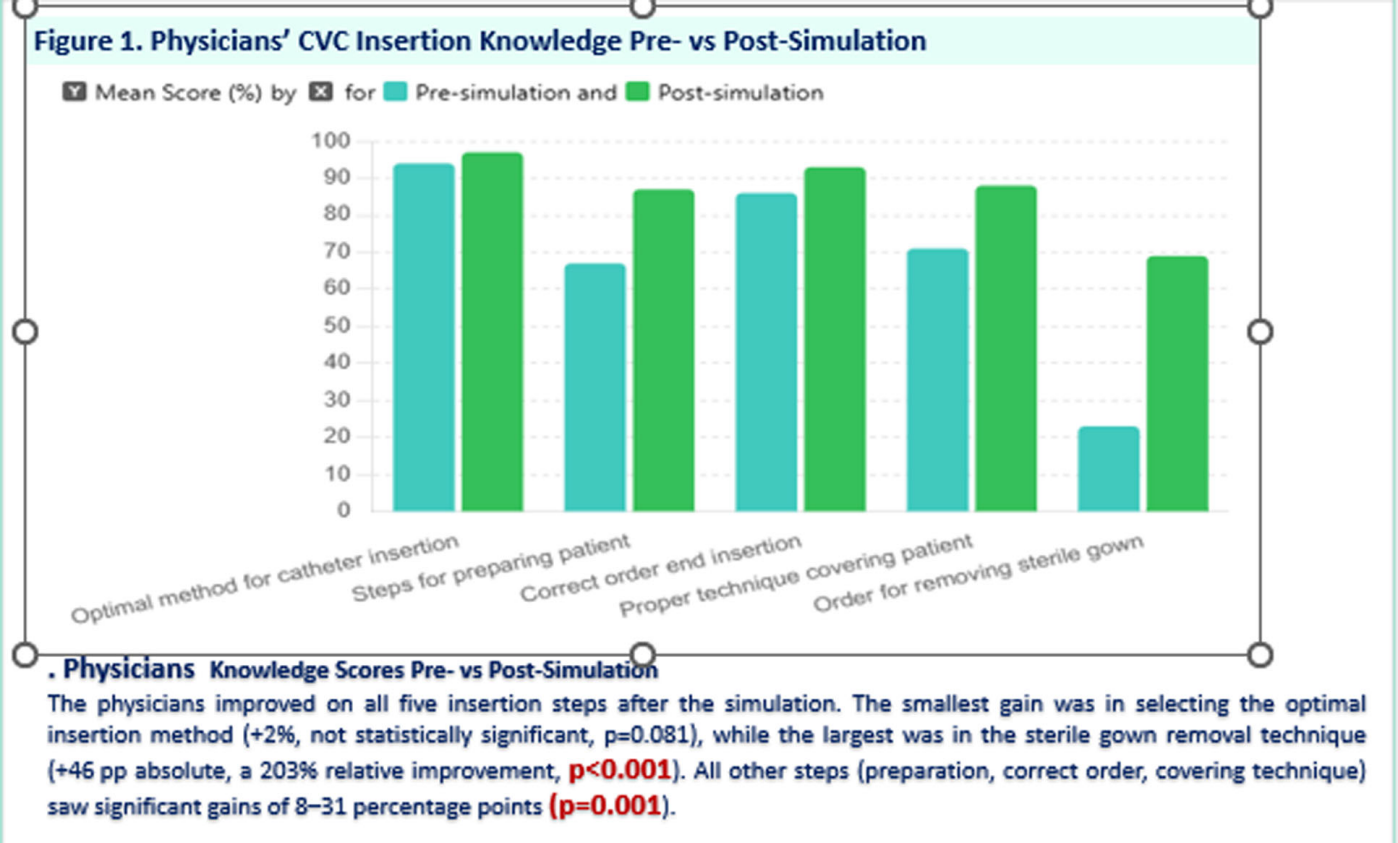

**Figure:**
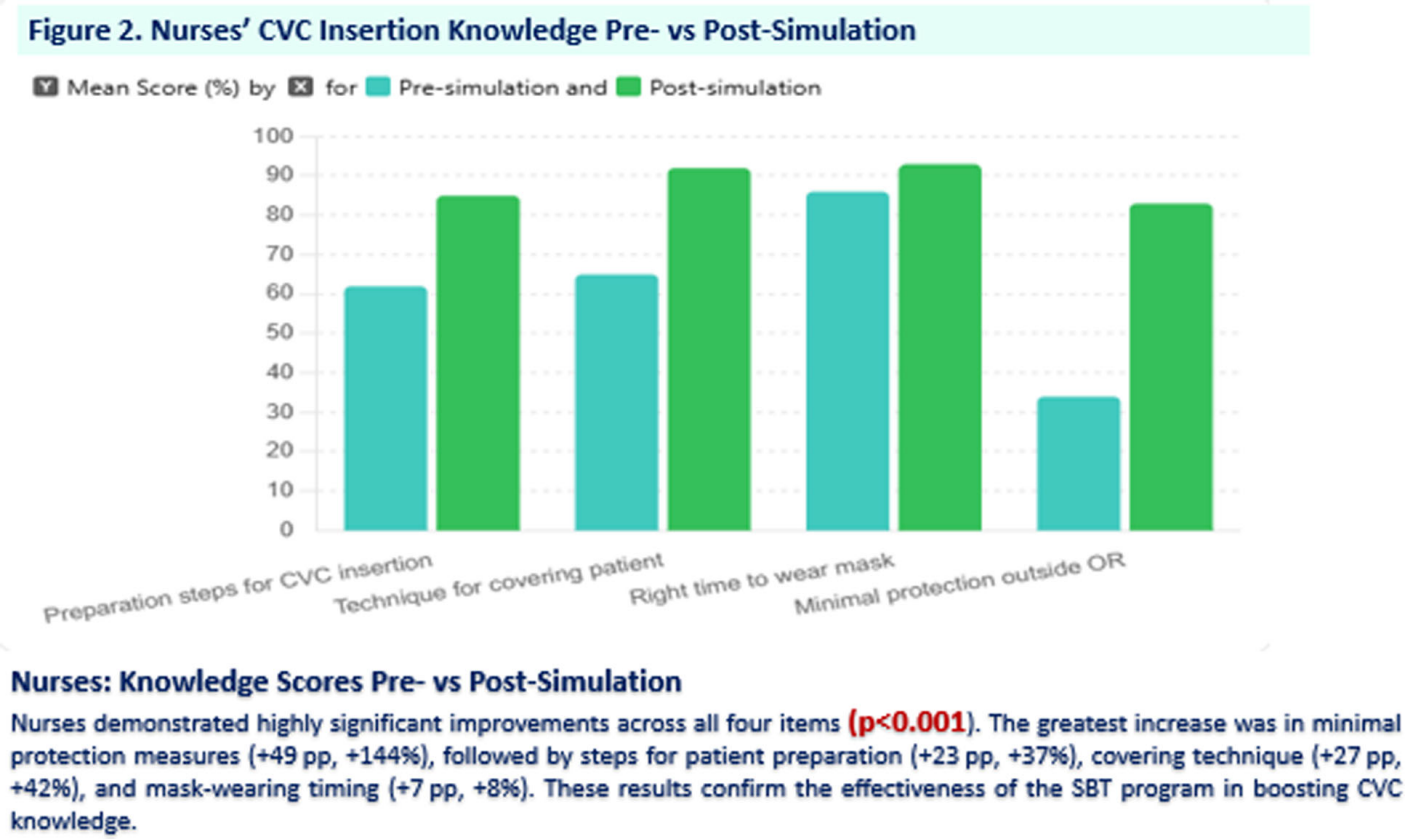

**Figure:**
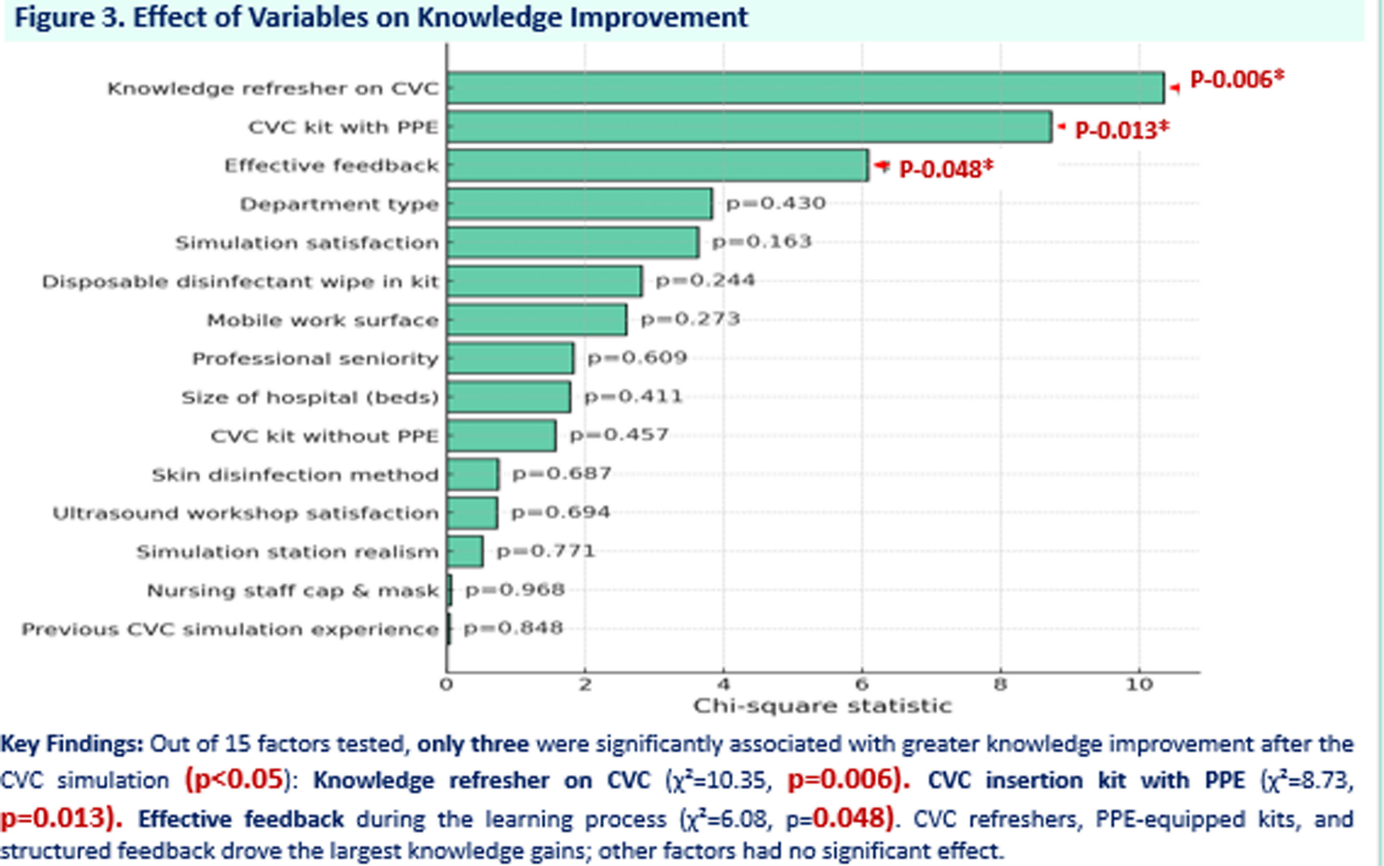

**Figure:**
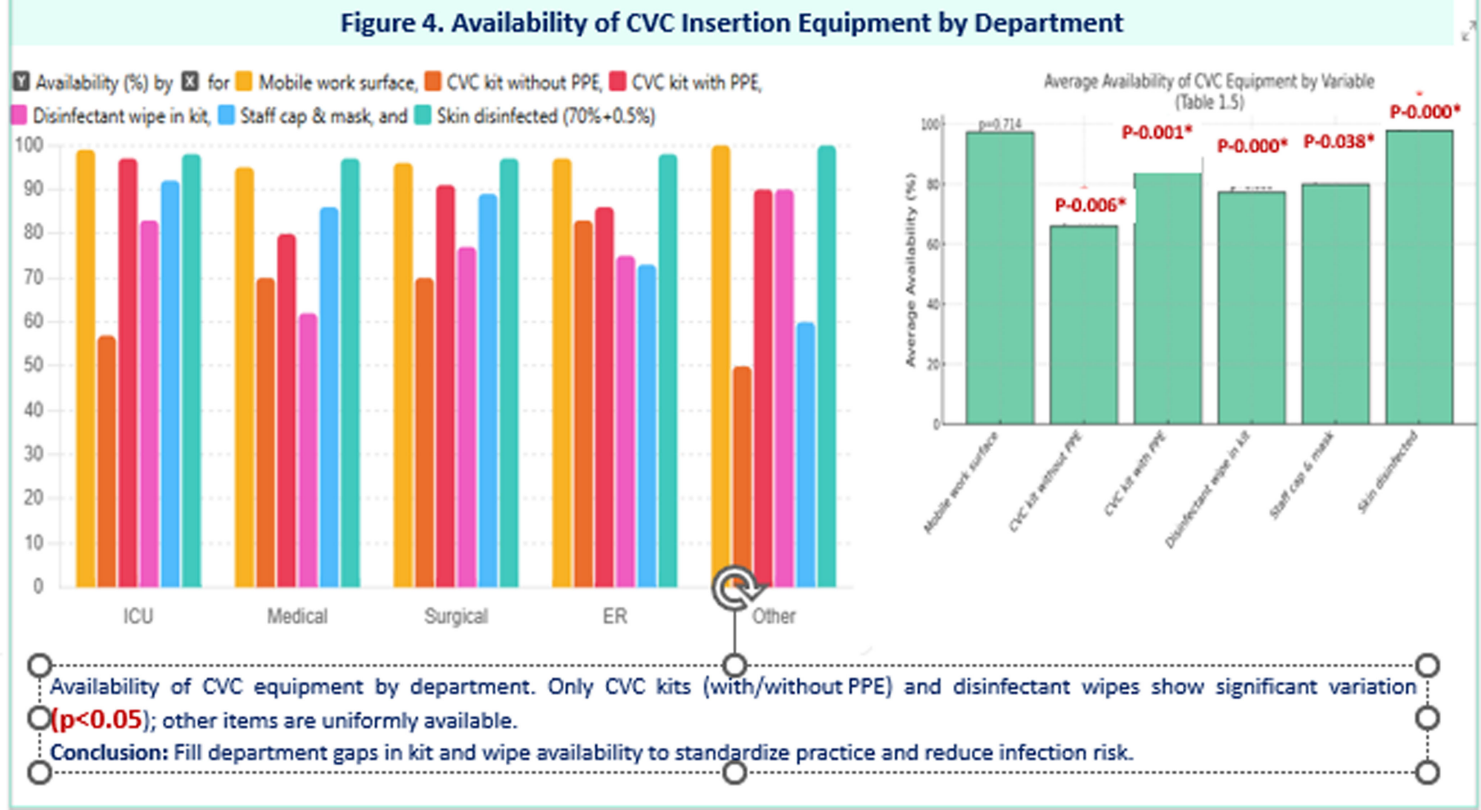

**Figure:**